# The Effects of Feedback on Children's Engagement and Learning Outcomes in Robot-Assisted Second Language Learning

**DOI:** 10.3389/frobt.2020.00101

**Published:** 2020-08-04

**Authors:** Mirjam de Haas, Paul Vogt, Emiel Krahmer

**Affiliations:** ^1^Department of Cognitive Science and Artificial Intelligence, Tilburg School of Humanities and Digital Sciences, Tilburg University, Tilburg, Netherlands; ^2^Department of Communication and Cognition, Tilburg Center for Cognition and Communication, Tilburg School of Humanities and Digital Sciences, Tilburg University, Tilburg, Netherlands

**Keywords:** child-robot interaction, second-language learning, robot tutor, feedback, engagement, preschool children

## Abstract

To investigate how a robot's use of feedback can influence children's engagement and support second language learning, we conducted an experiment in which 72 children of 5 years old learned 18 English animal names from a humanoid robot tutor in three different sessions. During each session, children played 24 rounds in an “I spy with my little eye” game with the robot, and in each session the robot provided them with a different type of feedback. These feedback types were based on a questionnaire study that we conducted with student teachers and the outcome of this questionnaire was translated to three within-design conditions: (teacher) preferred feedback, (teacher) dispreferred feedback and no feedback. During the preferred feedback session, among others, the robot varied his feedback and gave children the opportunity to try again (e.g., “Well done! You clicked on the horse.”, “Too bad, you pressed the bird. Try again. Please click on the horse.”); during the dispreferred feedback the robot did not vary the feedback (“Well done!”, “Too bad.”) and children did not receive an extra attempt to try again; and during no feedback the robot did not comment on the children's performances at all. We measured the children's engagement with the task and with the robot as well as their learning gain, as a function of condition. Results show that children tended to be more engaged with the robot and task when the robot used preferred feedback than in the two other conditions. However, preferred or dispreferred feedback did not have an influence on learning gain. Children learned on average the same number of words in all conditions. These findings are especially interesting for long-term interactions where engagement of children often drops. Moreover, feedback can become more important for learning when children need to rely more on feedback, for example, when words or language constructions are more complex than in our experiment. The experiment's method, measurements and main hypotheses were preregistered.

## 1. Introduction

A recent trend in education is to have social robots take on the role of educational tutors to support, for example, second language learning (Westlund and Breazeal, 2015; Belpaeme et al., 2018; Vogt et al., 2019). In educational settings, learning a (second) language typically involves social interactions between the child and the teacher. During these interactions, children constantly receive feedback about their performance. It has been shown that human feedback can have a clear impact on children's learning process and outcomes (Wojitas, 1998; Hattie and Timperley, 2007). Feedback is therefore an important part of the social interactions that facilitate language learning, which begs the question what the impact of various feedback types is when feedback is provided by a robot rather than a human.

Throughout many years researchers have investigated how (human) feedback can have an influence on second language learning. Focusing on children learning a second language, research has shown that receiving feedback benefits children's language development more than receiving no feedback (Mackey and Silver, 2005). Moreover, different types of feedback can help children in several ways. You can, for example, use positive feedback to reward and motivate children when they are correct, or use negative feedback to correct children's language and thereby improve children's learning gain (Hattie and Timperley, 2007).

While there have been many studies about robots for educating children, only few of these have investigated the effects that different types of feedback can have on children's engagement and learning performance (Gordon et al., 2016; De Haas et al., 2017; Ahmad et al., 2019). Usually, studies design feedback strategies for robot tutors based on results from educational studies involving only humans without investigating the effect that these strategies have on children's engagement and/or performance (e.g., Mazzoni and Benvenuti, 2015; Westlund and Breazeal, 2015; Gordon et al., 2016; Kennedy et al., 2016). However, it is not evident that the effect of human strategies will be the same when a robot uses them, because a robot has substantial cognitive and physical limitations compared to a human. For example, robots cannot produce the same facial expressions as humans or humans' subtle cues, thus are limited in providing facial cues that humans use to provide non-verbal feedback.

One recent study manipulated non-verbal and verbal feedback based on the child's emotional state (Ahmad et al., 2019). Results showed that children's engagement over time remained relatively high and children's word knowledge increased over time with positive or neutral feedback. While their results suggest that robot feedback can have a positive effect on children's engagement and learning gain, they did not compare different variations of positive and negative feedback or compared it against no feedback.

The results of Ahmad et al. (2019) are consistent with findings from human studies and demonstrate that feedback does not only enhance children's language performance, but also engages children. Positive feedback engages because it validates children's answers and thus boosts their confidence (Henderlong and Lepper, 2002; Zentall and Morris, 2010). Similarly, negative (corrective) feedback corrects and teaches the child the correct word which could result in a motivated child. However, both positive and negative feedback can also decrease engagement. On the one hand, too many repetitions of positive feedback can become meaningless for a child and can result in less intrinsic motivation (Henderlong and Lepper, 2002; Boyer et al., 2008). On the other hand, negative feedback can decrease the child's confidence and thereby decrease the engagement between the teacher and child (Wojitas, 1998).

Consequently, if used correctly, feedback can result in increased learning gains. Children become more intrinsically motivated by positive feedback, which increases the children's interest and their task engagement and therefore their skills. These increased skills will motivate the children further and engage the children to a greater extent (Blumenfeld et al., 2006).

This paper describes a study that investigated how preschool children respond to different types of feedback provided by a robot tutor. In the experiment, children interacted with a humanoid robot tutor in three different second-language sessions, and in each session the children received a different type of feedback. These types of feedback were designed based on a survey among student teachers, resulting in a strategy preferred by these student teachers, a strategy dispreferred by them and a strategy using no feedback at all. We analyzed the effect of these different types of feedback on the children's task engagement and learning gain over time.

## 2. Background

### 2.1. Feedback

Numerous studies have shown that feedback facilitates second language learning (Lyster and Ranta, 1997; Henderlong and Lepper, 2002; Long, 2006; Hattie and Timperley, 2007). It can help to improve pronunciation, word-choice and grammar, and makes it easier for children to understand what is correct or incorrect in the foreign language. Feedback is not only used to correct children, but for example also by teachers to contribute positively to children's own feeling of competence and success and therefore encourage children to continue with a task (Blumenfeld et al., 2006; Hattie and Timperley, 2007). The type of feedback provided, however, matters (Shute, 2008). You can, for example, provide explicit negative feedback by indicating that something is wrong with children's answers, but without specifying what was wrong (e.g., “That's wrong.”). It is also possible to provide corrective feedback by correcting children's answers or hinting toward it (e.g., “You said runned, but it should have been ran” or “it should not have been runned, but.?”). Prompting children with an extra attempt (“Try again.”) is an implicit way of saying something was wrong. Hattie and Timperley (2007) propose a combination of these three types as good way of providing feedback. The combination provides children with explicit notions where the mistake was made, what went wrong and makes them to try again. Nevertheless, sometimes separate feedback is also sufficient. For example, using explicit negative feedback (i.e., stating explicitly that something is wrong) seems to be most beneficial for children who are struggling with a task, such as novel learners (Kluger and DeNisi, 1996; Shute, 2008).

Teachers, however, mostly provide negative feedback implicitly by using recasts (i.e., a type of feedback in which the teacher repeats the incorrect phrases in a correct form), but they still try to make sure that children reach their goal (Lyster and Ranta, 1997; Long, 2006). Although these recasts have been found to be used more often than the other feedback types, they seem to be less effective in helping the learner to reach their learning goal. Lyster and Ranta (1997) investigated the role of negative feedback and found that when teachers explicitly mentioned the fact that an error was made in their negative feedback, it led to a higher learning gain than when they did not, which suggests that explicit negative (or corrective) feedback can be more effective than implicit feedback by using recasts.

Feedback is not always negative or corrective, it can also be positive. In general, teachers mostly use positive feedback explicitly (praise) and not implicitly (Hattie and Timperley, 2007). The advantage of praise is that it approves children's answers and makes the task encouraging and motivating (Henderlong and Lepper, 2002). When children receive positive feedback, they become happy, and are therefore more committed and intrinsically motivated to complete a task. However, there are also downsides to providing positive feedback. When children receive too much positive feedback, they rely on the feedback and will not learn when they do not receive the feedback anymore (Henderlong and Lepper, 2002). In addition, when the use of praise is non-specific or ambiguous, such as saying “good job” or “beautiful” makes children not understand what part of their answer elicited the feedback and they will not know how to respond (Hamilton and Gordon, 1978). Thus, positive feedback should refer to the learning task and at the same time remain motivating enough in order to be effective.

#### 2.1.1. Feedback, Engagement, and Learning

Engagement seems to have a positive effect on language learning (Christenson et al., 2012). A considerable amount of studies have shown that robots are engaging interaction partners for both adults and children (see for an overview Kanero et al., 2018). Engagement normally starts high due to the novelty effect but then seems to decrease over time (Kanda et al., 2007; Westlund and Breazeal, 2015; Rintjema et al., 2018). When talking about engagement, it can be helpful to distinguish between two kinds of engagement: robot-engagement, referring to how engaged a child is with the robot, and task-engagement, which focuses on how engaged a child is with the learning task. Clearly, these are not necessarily the same: a child can be very engaged with their social partner, the robot, but not with the task, or visa versa. Moreover, the effect of these different engagement types on learning gain can differ. For example, one study by Kennedy et al. (2015) used a highly engaging robot partner and, as a result, children were so distracted by the robot that they focused less on the task and therefore learned less. In their study, children who were highly engaged with the robot, learned less instead of more while it is possible that children who are highly engaged with the task, will still learn more. Consequently, it is useful to measure both types of engagement: task-engagement and robot-engagement.

Research in HRI has looked at many ways of keeping general engagement high, but did not investigate the role that different types of feedback could play here. For example, Ahmad et al. (2019) looked at the role of adaptive feedback on the children's emotion on engagement, but they did not investigate the effect of different types of feedback.

Feedback, however, can have an influence on children's motivation and their self-evaluation (Zentall and Morris, 2010), which—in turn—can influence engagement. Blumenfeld et al. (2006) suggested a feedback loop: in order to increase children's engagement, children first have to be motivated, which will then increase their interest in the task, which in turn will engage children followed by the children's learning gain. When children improve their language skills, this can lead to even higher motivation and further result in a higher engagement.

The influence of feedback on motivation depends on the type of feedback. For instance, praise that is specifically linked with the children's effort (e.g., “You are a good drawer” after drawing a picture) motivates children more than other types of praise, even when only 75% of the praise is linked with effort (Zentall and Morris, 2010). Moreover, Corpus and Lepper (2007) showed that for preschool children all praise enhanced motivation when they compared it with neutral feedback (“OK”). They compared motivation of preschool children with older children, and found that only for older children (fourth and fifth graders) the type of praise had an influence on their motivation, while preschool children benefited from all feedback equally. Another study found similar results: Morris and Zentall (2014) measured ambiguous praise (“Well done!”, “Yeah,” “Awesome”) and found higher persistence, higher self-evaluations and fewer fixations on later mistakes. Apparently, children interpret ambiguous praise in the most beneficial manner for themselves. However, they also found that the use of gestures (“Thumbs up” and “High five”) resulted in the highest self-evaluations.

The reason why feedback has an influence on motivation and therefore engagement can be explained by the Self-Determination Theory (Deci and Ryan, 1985). This theory poses that learners continue a task longer when their motivation is based on intrinsic aspects, such as pleasure and satisfaction, compared to when motivation is based on external rewards (Deci and Ryan, 1985). This intrinsic motivation arises particularly when a task contains autonomy and competence and is strengthened by a sense of relatedness between learner and teacher (Ryan and Deci, 2000). For example, autonomy increases when a learner can choose themselves what kind of activity to do, or when he or she receives informative rewards and non-controlling instructions. A higher degree of autonomy leads to increased intrinsic motivation and, in turn, higher levels of engagement. Moreover, competence increases with praise (Blanck et al., 1984), because it enhances the children's feeling of being capable to successfully complete a challenging task. Competence, especially in combination with autonomy, plays a considerable role in retaining intrinsic motivation. There are also disadvantages of praise, for example, when children first receive praise but are not able to successfully complete the task, their motivation can decrease (Zentall and Morris, 2012). Moreover, too much positive feedback can decrease the children's own curiosity (Henderlong and Lepper, 2002).

Negative feedback has been found to decrease intrinsic motivation, specifically the feeling of competence (Deci et al., 1991). It can potentially decrease children's self-efficacy or their active participation and engagement in the learning task, because children become unmotivated when receiving negative feedback (Wojitas, 1998). On the other hand, negative feedback can also have a positive influence on motivation, as it can help children to understand what they are trying to learn and to correct their mistakes (Hattie and Timperley, 2007). Kluger and DeNisi (1996) suggest that, similar as with praise, the effect of feedback is not only dependent on a link between behavior and feedback, but also on how the feedback was provided and how the learner interprets the feedback.

The combination of praise and negative feedback can be challenging enough for children, but at the same time motivates children enough to want to continue with the task. For example, if children additionally receive negative feedback to correct their mistakes and hear praise when they correctly answer a question, this can enhance the effect of both feedback types. Summarizing, feedback has the potential to both engage and disengage children (Dempsey and Sales, 1993), depending on the type of feedback given. Feedback (especially praise) can increase the intrinsic motivation of children, which increases their engagement. Engaged children are more motivated, learn faster, will be more likely to complete the task and to repeat the task, which leads to a better result (Dörnyei, 1998). However, it is not clear yet whether the rules that apply to human teacher-child interactions also apply to robot-child interactions.

#### 2.1.2. Feedback in Child-Robot Interaction

Studies with educational robots for children that have explicitly looked at the role of feedback are sparse. While many studies have incorporated the use of feedback, specifically praise (Mazzoni and Benvenuti, 2015; Westlund and Breazeal, 2015; Gordon et al., 2016; Kennedy et al., 2016), they did not test the effect of feedback on the children's engagement or learning gain nor the effects that different forms of feedback may have. These studies investigated the role of praise either by incorporating it as part of a robot's strategy (Westlund and Breazeal, 2015; Kennedy et al., 2016), by looking at specific responses of children on occurrences of praise (Serholt and Barendregt, 2016) or on the effect of timing of the praise (Park et al., 2017). It seems that children notice the praise and react to it, however, these studies did not investigate its direct effect on engagement and learning gain. For example, Kennedy et al. (2016) compared a high verbal availability robot and a low verbal availability robot. The high verbal availability robot used—among other social behaviors—more expressive praise than the low verbal availability robot. Children of approximately 8 years old practiced different French grammar rules with one of the robots. The authors found no significant difference in learning gain for the robot that used more expressive positive feedback, but the children reported to have noticed the praise and payed attention to it.

In another study, Serholt and Barendregt (2016) investigated children's responses to the robot's praise. In their long-term study, the robot gave praise on the children's performance of the previous session. Positive feedback did not go unnoticed, 70% of the children acknowledged the robot during feedback through verbal or gestural responses such as smiling. Similarly, Park et al. (2017) explored whether the timing of a robot's praises would influence the engagement of children. Children had to tell a robot a story and the robot reacted on their emotional level as a form of feedback. For example, when children had a high energy level, the robot played a large excited motion. Park and colleagues compared two conditions, one with a robot that reacted every 5 s on the child without changing its energy level, and one with a robot that reacted during breaks between child speech and changed the energy level of its responses appropriately. The children seemed to be more engaged with latter robot that changed its feedback to their energy level. Likewise, Westlund and Breazeal (2015) used a non-humanoid robot to teach children a second language and found that children learned with a social robot more than with a non-social robot. Both robots used positive phrases when children were correct, e.g., “Good job!” or “You're working hard!” and only provided hints with an incorrect answer, e.g., “I think it was that one.” However, the social robot added expressive phrases based on the child's emotional state (e.g when children were excited, the robot first reacted with “woo hoo” before the feedback).

While many robots use praise, which is an explicit form of positive feedback, explicit negative feedback is not often used in child-robot studies. Typically, studies incorporated implicit feedback by using hints (e.g., “I think it was the other one,” Gordon et al., 2016) or by introducing doubts (“Are you sure?” Mazzoni and Benvenuti, 2015).

Three studies that specifically investigated the effect that feedback has on learning and/or engagement are those by De Haas et al. (2017), Resing et al. (2019), and Ahmad et al. (2019). De Haas et al. (2017) conducted a between-subject study with 4-year-old pre-school children that compared the effect that three different feedback strategies (peer-like, adult-like, and no feedback) had on learning gain and engagement. The feedback strategies did not affect the learning gain or the engagement measured through eye-contact. Instead, children showed a substantial amount of individual differences in how they engaged with the robot across the three feedback conditions. Some children focused completely on the robot, while other children focused more on the researcher by asking for more guidance. Even though children did not seem to benefit from the different types of feedback, this study consisted of only one session which—due to the novelty effect—may have disturbed the effect that different forms of feedback may have.

Resing et al. (2019) reported a study where 6 till 9-year-old children had to solve a puzzle together with an owl-like robot that either helped them by giving feedback or did not provide any help. The help-providing robot used both verbal and non-verbal feedback. It shook its head and had blinking eyes when their answer was incorrect as a way of providing non-verbal (explicit) negative feedback, or nodded and said “Well done!”, with (different) blinking eyes as a form of explicit positive feedback. Children trained by the robot with feedback became better in solving new puzzles than children trained with the other robot. However, again, children showed large individual differences in the number of corrections they needed.

Ahmad et al. (2019) addressed individual differences between children and compared in a between-subjects design a robot that adapted its feedback with one that did not. They studied how children between 10 and 12 years old responded to the robot's feedback during 2 weeks. The robot adapted its feedback behavior to the children's emotional state. For example, when children were rated as happy the robot used that in its feedback (“You are looking happy, and I'm happy that you are in front of me. Let's learn another word”). During the game, the robot kept referring to the game outcome, only in the post-test the robot provided feedback on learning performance (“I am happy that you got it wrong in session one, but this time your answer is correct' or ‘It's sad that you didn't remember this word, the correct answer is.”). Ahmad and colleagues found that the children's engagement remained relatively high (or stable) when interacting with the adaptive robot, while their engagement lowered over time with the non-adaptive one. Moreover, children's learning gain was higher with the adaptive robot, compared to the non-adaptive one. While these results are promising, this study did not investigate the effect of different feedback strategies.

Generally, developers of robot tutors base the educational strategies of the robot on the already existing human studies and use those strategies in their child-robot interactions without studying whether these strategies are similarly effective. Most child-robot studies use praise as a motivator in their experiments and are hesitant to use explicit negative feedback. It is not clear what type of negative feedback works best for robots, although in educational studies it seems that mentioning the children's mistake seems to be more effective for language learning. In this paper, we address this gap in knowledge by investigating the effect of different forms of feedback on both task-, robot-engagement and learning gain.

### 2.2. Teachers' Feedback

In preparation of the present study, we carried out a survey among student teachers concerning their views on how a robot should provide feedback. The aim of this survey was two-fold: (1) To gain insights how student teachers' would think the robot should provide feedback to children giving correct and incorrect answers in a tutoring setting, and with varying levels of the children's engagement at the time feedback is given. (2) To create a data set with different feedback phrases that student teachers would use. We interviewed student teachers instead of practicing teachers, because students are more likely to work with technologies in the future, such as social robots, than teachers who already worked for many years. Moreover, receiving many responses was more feasible with student teachers than with teachers.

In our survey, we showed 27 student teachers 40 video fragments of both engaged and disengaged children interacting with a robot in a second language tutoring experiment reported in De Wit et al. (2018). All fragments showed a robot teaching 5- to 6-year-old Dutch children animal words in English as a second language. In each fragment, the robot expressed an English word and asked the child to select—on a tablet—the animal he or she thought that the word referred to. The fragment ended right after the child answered to this request. After watching each fragment, the student teachers were asked to provide a feedback suggestion. The survey was carried out in a between-subject design with two conditions: in one condition (closed questions), student teachers could choose between six feedback strategies (three positive and three negative), and in the other condition (open-ended questions) they could freely write the feedback themselves. This closed questions survey would provide insights of what strategy student teachers would choose, and the open questionnaire would create a data set of different feedback phrases.

We did not find a difference between student teachers' suggestions for engaged or disengaged children. However, we found that the suggested forms of feedback differed substantially between the closed and open-ended questionnaires: In the closed questions survey, the majority of the student teachers chose to use an explicit positive phrasing together with an explanation in the form of a translation [“Goed zo! Een ‘hippo’ is een nijlpaard” (Dutch)—“Well done! A ‘hippo’ is a hippo” (English)], and they chose a correction of the child's answer through repetition and translation of the target words [“Een hippo is een nijlpaard, je moet de nijlpaard aanraken” (Dutch)—“A ‘hippo’ is a hippo, you have to touch the hippo” (English)] as a means of providing implicit negative feedback.

In the case of the open-ended survey, the student teachers chose for both positive and negative feedback to only provide an explicit phrasing without repeating the target words for both positive feedback [“Goedzo” (Dutch)—“Well done” (English)] and negative feedback [“Helaas dat was niet goed” (Dutch)—“Unfortunately, that was not correct” (English)]. Moreover, we found that in the open-ended questionnaire student teachers varied their phrasing of the feedback considerably. These results indicate that student teachers do not have a straightforward strategy for choosing how to provide feedback.

After the surveys were analyzed, we discussed the findings with a subset of the student teachers. They suggested two main reasons why these results differed. Firstly, correction and explanation (e.g., through repetition of target words) is essential for negative feedback. This was the main reason why they chose to repeat the target words in the closed-ended questionnaire. Secondly, they indicated that variation in the form by which feedback is provided is also crucial. The robot should not repeat the same phrase throughout the whole lesson. Student teachers participating in the open-ended questionnaire focused more on creating varying feedback phrases and less on the repetition of the target word.

Based on these findings, we concluded that the “preferred” feedback strategy would combine the results from the closed questions survey with the open-ended survey: take an explicit feedback phrase (e.g., “Well done” or “That's wrong”), add a repetition of the target word, and provide children an extra attempt when their answers are incorrect. Since variation is key, the feedback phrases should vary, based on the data set created by the open-ended survey.

### 2.3. This Study

The present study investigates whether 5- and 6-year-old children are more engaged with the task and with the robot, and learn more words when participating in a second language (L2) training with a robot that provides feedback as recommended by the student teachers (preferred feedback), compared to a robot that provides feedback contrary to what was recommended by the student teachers (dispreferred feedback), and compared to a robot that provides no feedback at all (no feedback). As our survey with student teachers revealed, providing adequate feedback is a complex matter that consists of multiple strategies, which are hard to separate, thus making it difficult to investigate such individual factors experimentally. We therefore decided to combine multiple factors in our preferred and dispreferred feedback strategies, and explore to what extent these strategies, as performed by a robot, influence children's engagement and learning gain in an L2 tutoring scenario.

Every child receives three sessions with different robots, each providing a different form of feedback, thus allowing us to investigate how children react to the different forms of feedback using a within-subjects design. We based the training sessions on previous studies in which children played an “I spy with my little eye” game with a NAO robot to learn different L2 words (De Wit et al., 2018; Schodde et al., 2019).

Based on previous findings in literature regarding the role of feedback in second language learning, and previous studies that address feedback in child-robot interactions (Ahmad et al., 2019), we hypothesize that children will be more task- and robot-engaged when receiving (either preferred or dispreferred) feedback than when they do not receive feedback (H1a). Especially positive feedback is expected to increase the children's intrinsic motivation for the task and thus their engagement. We also hypothesize that children will remember more words when receiving feedback than when receiving no feedback (H1b). Feedback can help to understand whether an answer is correct or not and may indicate what the correct form should be, thus providing insight into the learning process and helps to improve the learning performance.

Moreover, we hypothesize that children will be more task- and robot-engaged with (H2a) and will remember more words from (H2b) a robot that provides feedback as preferred by a student teacher compared to a robot that provides dispreferred feedback. When feedback is varied (as in the preferred feedback strategy), children are expected to pay more attention to it, boosting their confidence and with that their task-engagement. The varied feedback of the robot can additionally increase the children's interest in the robot and with that their robot-engagement. In contrast, when a robot repeatedly uses the same phrase as feedback (dispreferred feedback), children might get tired of this repetition and as a result will pay less attention to the robot. Additionally, children can practice with the preferred feedback once more in the case of a mistake and thus improve their knowledge, which they cannot with the dispreferred feedback strategy and which might lead to an increase in their task-engagement. Moreover, the preferred feedback also provides children with an explicit notion where the mistake has been made, what went wrong and how they can fix it by trying again (the three rules of good feedback according to Hattie and Timperley, 2007).

## 3. Methods

The research questions, hypotheses and analyses in this study have been preregistered at AsPredicted^1^ and the source code has been made publicly available^2^.

### 3.1. Design

The study was a within-subjects design, where all participants were assigned to all feedback strategies/conditions (each session a different strategy). The strategies for providing feedback were based on the survey asking student teachers how they would make the robot provide feedback in situations comparable to the ones in this experiment, translating to a preferred strategy and dispreferred strategy. The order of the feedback strategies and word sets were counterbalanced using a 3 × 3 latin-square to reduce an order effect. The three strategies/conditions were

1. Preferred feedback
2. Dispreferred feedback
3. No feedback

**Figure 1:**
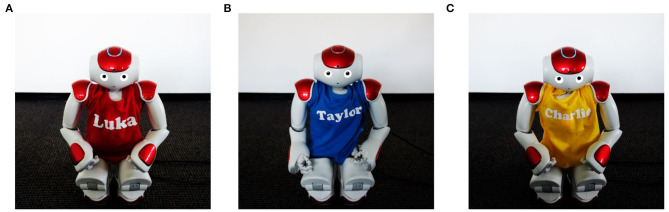
**(A–C)** show the different shirts for each sessions. All children saw the robot wearing the red shirt during the first session and all children saw the robot wearing the yellow shirt during the last session.

Each child received three sessions with the robot, and could learn 18 words in total and 6 in each session. In all conditions, all sessions were the same, except for the words learned, the feedback strategy that the robot used and the shirt the robot was wearing (to give the impression that children were playing with three different robots, see Figure 1).

### 3.2. Participants

In total, 72 native Dutch-speaking children aged 5 and 6 years participated in the current study. The participants were recruited from three elementary schools located in the Netherlands. Bilingual children were excluded from the study. A pre-test showed that 12 children were familiar with more than half of the target words and these children were excluded from the study in accordance with the exclusion criteria of our preregistration. Furthermore, four children dropped out of the study for various reasons like unwillingness to continue (3) or sickness (1). This resulted in 56 children (28 boys, *Mage* = 5 years and 6 months, *SDage* = 5 months) participating in the final experiment. All parents gave informed consent for the participation of their child.

### 3.3. Materials

The Softbank Robotics NAO robot and a Microsoft Surface tablet computer were used. The lessons involved one-on-one interactions between robot and child. We did not rely on automatic speech recognition because speech recognition has been shown to not work well with this age group (Kennedy et al., 2017). Instead the experimenter used a Wizard of Oz technique when the child had to say something to the robot in the beginning of the experiment. The robot was placed in a crouching position in an angle of 90 degrees next to the sitting child to give the robot the same perspective of the child, while still being able to face the child. The tablet was placed on top of a small box in front of the robot and child. A video camera placed on a tripod facing the child to record the child's responses and facial expressions. A second camera was placed from the side to get a more complete overview of the interactions. Each session was distinguished by a different color shirt and robot name (see Figure 1). We used the different shirts and names to make it known to children that they would play with three different robots, with different robot behaviors (namely the robot feedback strategies). The shirts were not linked to feedback conditions or different word sets, but rather to the lesson number. In other words, all children started with the robot wearing the red shirt called Luka during the first session and ended with the robot wearing the yellow shirt called Charlie.

#### 3.3.1. Target Words

**Figure 2:**
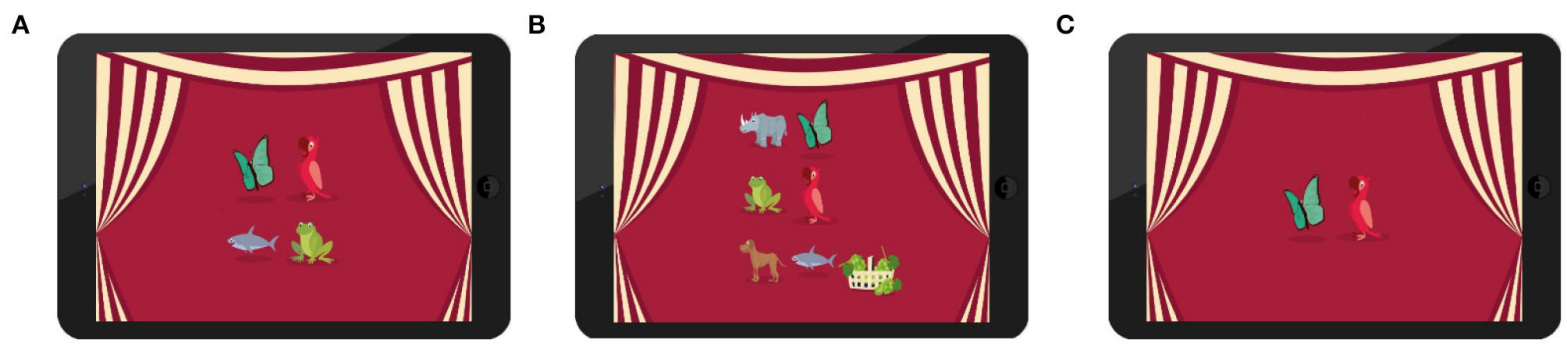
**(A)** Training rounds. Each round the robot named one animal that children had to find **(B)** In-game test. Children had to drag a grape to the animal that the robot named **(C)** second attempt after wrong answer. Children were allowed to correct themselves in the preferred feedback condition. In this example, the child wrongly chose a butterfly instead of a parrot and could correct his/her mistake by selecting the correct one.

In total 18 target words were selected and during each lesson, children learned six target words. Target words were selected such that children can be expected to have acquired those in their first language but arguably not in their second language. Moreover, we selected words that would not be too similar in their L1 and in their L2 [e.g., not “Olifant” (Dutch) and “Elephant” (English)]. All 18 words were divided in three word sets based on their frequency in the children's first language. We used the dataset of Schrooten and Vermeer (1994) and placed each word in a frequency bin. Words in the same bin were randomly assigned to the different word sets. For example, the word “dog” was from the same frequency bin as the words “bird” and “horse” and were thus added to different word sets. See Table 1 to see all target words with their frequency. We used cartoon-like images of the target animals during the experiment (see Figure 2 for examples).

**Table 1:** Target words with their frequency scores in Dutch taken from Schrooten and Vermeer (1994).

**Word set 1**			**Word set 2**			**Word set 3**		
**Dutch**	**English**	**Freq**	**Dutch**	**English**	**Freq**	**Dutch**	**English**	**Freq**
Hond	Dog	98	Vogel	Bird	72	Paard	Horse	64
Kikker	Frog	27	Kip	Chicken	30	Konijn	Rabbit	48
Vlinder	Butterfly	22	Nijlpaard	Hippo	16	Varken	Pig	36
Papagaai	Parrot	9	Slang	Snake	14	Eekhoorn	Squirrel	13
Haai	Shark	9	Slak	Snail	14	Zeehond	Seal	10
Neushoorn	Rhino	9	Walvis	Whale	9	Hert	Deer	9

*Words that have a higher score are more familiar to children in Dutch*.

#### 3.3.2. Pre-test

Before the children started the three sessions, we tested their L2 knowledge of the 18 target words with a comprehension test which was a picture-selection task. In this test, children were presented with a pre-recorded target word spoken by a bilingual speaker of Dutch and English and asked to choose which one out of four pictures matched this word [“Waar zie je een dog?” (Dutch) “Where do you see a: dog?” (English)]. The presentation of the target words in the pre-test was randomized for each child. We presented each target word one time during the pre-test.

#### 3.3.3. Post-test

The children's long-term knowledge was tested between 2 and 3 weeks after the last session with the comprehension test. The test was the same as the pre-test only this time, each target word was presented three times in a random order to reduce chance level performance due to guessing. The reason for not doing so in the pretest was to reduce the chance of children learning from this task (Smith and Yu, 2008). A word was registered as correct if it was selected correctly at least twice out of the three trials. Additionally, we tested three different pictures of the animals in order to generalize the children's knowledge. To be more specific, we used a cartoon-like picture, a drawn picture (the same as in the experiment) and a photograph of the target animal.

In addition to the measurements described in this paper we also carried out a perception questionnaire of the robot at the end of all sessions. We will not discuss those results because this questionnaire is beyond the scope of this paper.

### 3.4. Tutoring Sessions

The lessons were based on the children's game “I spy with my little eye” and on the interaction described in Schodde et al. (2019). The whole interaction was in the children's L1, except for the target words. Before the three tutoring sessions, children had a group introduction to the robot and took a pre-test.

The tutoring session had four parts which were all repeated during all three tutoring sessions:

1. Start phase. The robot explained that he was a friend of the group introductory robot, he asked for the child's name, age and some questions about their favorite animals and games. The robot finished with saying that “I spy with my little eye” is his favorite game and that he wants to play that with the children. He then explained the rules of the game.
2. Concept binding of the target words. To teach children the target words, the tablet showed an animal on the screen, the robot said the L2 word with the L1 translation and asked the child to repeat the word [e.g., “Een vogel is een bird in het engels, zeg mij maar na bird” (Dutch). “A bird is a *bird* in English, repeat after me *bird*” (English)]. Only after the child had repeated the animal, they continued to the next animal. When a child did not repeat the robot, the experimenter asked the child to listen to the robot and repeat after the robot. If a child was very hesitant to repeat the word, the experimenter would say it together with the child.
3. Training rounds. After the concept binding the robot explained to the child that he would ask for an animal and that the child had to search for it on the tablet screen. They first practiced with an L1 word that was no target (“Ik zie, ik zie wat jij niet ziet en het is een eenhoorn, zoek maar naar de eenhoorn,” “I spy with my little eye a unicorn, please search for the unicorn”). For each target word the tablet showed the target animal with three distractors (see Figure 2A). After the L1 practice round, the robot and child also practiced once in L2. After these two practice rounds they started the training of the target words. The robot constantly asked the child to search for a target word (“Ik zie, ik zie wat jij niet ziet en het is een < target word> zoek maar naar de < target word>, ‘I spy with my little eye a < target word>, please search for the < target word >”). Depending on the condition the robot provided feedback or not and the child continued to the next animal. There were 24 rounds in total, each animal was trained four times, which made the L2 exposure to all animals ten times in total for all conditions (twice in the concept binding, eight times during the practice rounds).
4. In-game test. After each session there was an in-game test that tested the short-term memory of the target words. The tablet screen showed all animals of that tutoring sessions and a bucket of grapes (see Figure 2B). Each round, the robot named an animal and the child had to feed this animal with one of the grapes. The robot asked the animals in random order and after each round the order of presenting the animals on the screen was shuffled.

All conditions had the exact same design, meaning that the lesson structure was the same, the tablet output was the same and the behavior of the robot was the same, except for the feedback. In all conditions, the robot used the standard following-gaze feature of NAO.

### 3.5. Feedback Conditions

All feedback was provided in the children's L1 to keep the L2 exposure consistent between conditions. A comparison of the different types of feedback can be found in Table 2. The feedback conditions were based on the student teachers' preferred response for the robot (preferred feedback), the opposite (dispreferred feedback) and a control condition was added where the robot did not use any feedback. Preferred and dispreferred feedback different on multiple aspects:

1. Variation. The robot used a variety of positive and negative feedback in the preferred feedback condition and no variation in the dispreferred feedback condition. We based the phrases on the student teachers' open-ended survey and can be found in Table 3. The robot randomly chose between six verbal phrases for positive feedback and negative feedback and the same phrase was never used twice in a row. We only added variation to the preferred strategy because the student teachers considered this crucial.
2. Extra attempt. The robot let children to try again after an incorrect answer in the preferred feedback condition and not in the other conditions. This was based on the student teachers' closed-ended answers where they relied heavily on the answer with the extra attempt. During the extra attempt, the tablet would only display the correct target word and the children's incorrect answer to help the children distinguish the two answers (see Figure 2C). After children correctly answered their second attempt, they received positive feedback.
3. Repetition. In the preferred condition, the robot would repeat the target word, either in addition to positive feedback or in addition to noting the mistake including the child's wrong answer. However, this was only done in 50% of all feedback to reduce the amount of repetition and because the student teachers did not always use a repetition (only in the closed-ended questionnaire and not in the open-ended questionnaire). The robot would only repeat the target word in the children's L1 (i.e., Dutch) to keep the amount of L2 exposure consistent over all children and to only focus on the effect of feedback.
4. Non-verbal feedback behavior. The robot used some non-verbal behavior when the child was correct in the preferred feedback condition, but not in the dispreferred feedback condition. This non-verbal behavior consisted of the robot nodding and displaying a rainbow colored pattern in the LED-eyes to indicate happiness.

**Table 2:** An example of the robot's feedback in the different feedback conditions.

	**Correct answer**		**Incorrect answer**	
**Condition**	**Dutch**	**English**	**Dutch**	**English**
Preferred	Goed gedaan, het was een vogel.	Well done, it was a bird.	Helaas, je hebt een vogel aangeraakt. Laten we het nog eens proberen!	Unfortunately, you selected a bird. Let's try again!
Dispreferred	Goed gedaan.	Well done.	Helaas, dat is niet goed.	Unfortunately, that was not correct.
No feedback	-	-	-	-

**Table 3:** The preferred feedback utterances.

**Positive**		**Negative**	
**Dutch**	**English**	**Dutch**	**English**
Goed gedaan!	Well done!	Helaas dat was niet goed.	Unfortunately, that was not correct.
Knap hoor.	Impressive.	Sorry deze is niet goed.	Sorry but this is not correct.
Ja goed gedaan!	Yes, well done!	Helaas, probeer het nog een keer.	Unfortunately, try again.
Ga zo door!	Keep going!	Jammer, we proberen het nog eens.	What a pity, let's try again.
Super!	Great!	Ah jammer, denk nog even goed na.	Ah pity, think again.
Heel knap gedaan.	Really impressive.	Super goed geluisterd, maar dat was niet goed, probeer het nog eens.	You listened very well, but this was not correct, try again.

*The robot's feedback varied between six different options*.

After the feedback was provided (or after the child's answer in the no feedback condition), the game continued to the next target word.

### 3.6. Procedure

#### 3.6.1. Robot Introduction and Pre-test

One week before the experiment, the children participated in a group introduction to familiarize themselves with the robot. During this introduction, based on Vogt et al. (2017), children learned how the robot moves and how to talk to it, and they played a game where they had to imitate the robot and they danced together. Unlike the robots during the experiment, this robot was not wearing a shirt. After this group introduction the children carried out a pre-test on their prior English knowledge in one-on-one sessions, as explained in section 3.3.2.

#### 3.6.2. Experiment

At least 1 week after this group introduction and the pre-test, we started the first tutoring sessions with the children. Children participated in a quiet room away from their classrooms. After the child was collected from her or his classroom for the first session, he or she was told that he or she would play a game on a tablet with a friend of the introduction robot. This was repeated every new session so each child saw four “different” robots in total (one introduction robot and three robots in the tutoring sessions). When the child entered the room with the robot, the experimenter told the child to sit in front of the tablet next to the robot and started the experiment. After the child finished the 24 rounds of “I spy with my little eye” and the subsequent in-game post-test, the experimenter filled in a questionnaire with the child about the robot. When this questionnaire was completed the experimenter brought the child back to the classroom. This was repeated for three times with at least 1 day in between the different sessions.

The complete interaction was autonomous, except for the detection of children's speech when they repeated the target words as instructed. For detecting the child's speech, the experimenter would press a button on a control panel once the child had repeated the robot's utterance. The interaction was a one-on-one interaction, but the experimenter stayed in the same room to intervene when necessary. For example, when a child did not repeat after the robot, the experimenter would try to encourage the child to repeat after the robot. Moreover, when the child had a question, the experimenter would say that she did not know the answer and directed the child's attention back to the robot. In other cases, when a child had to go to the bathroom, the experimenter paused the experiment and walked with the child to the bathroom and back. The duration of each session was around 11 min (Preferred: *M* = 14 min, *SD* = 2 min, Dispreferred: *M* = 11 min, *SD* = 1.5 min, No feedback: *M* = 10 min, *SD* = 1 min).

#### 3.6.3. Post-test

Two weeks after the last lesson, the children were collected from the classroom once more for the post-test.

### 3.7. Engagement Coding and Analyses

#### 3.7.1. Engagement Coding

Engagement was determined by manual coding of half of the data. Before coding, the two raters followed a coding training and practiced with different videos. Each video was rated on a Likert scale from 1 to 9, with 1 being a low level of engagement and 9 being highly engaged. We measured *task-engagement* that includes the attention that the child payed to the robot as instructor, but also to the task displayed on the tablet screen. Children were fully engaged, when they were completely “absorbed” in the activity, were open for new information, were very motivated, enjoyed the task and wanted to play with the robot (Laevers, 2005). Additionally, we rated *robot-engagement* that measures the children's attention and interest at the robot as a social interaction partner. Children were fully engaged with the robot, when they were interacting with the robot as a social conversation partner.

The coding scheme was based on the ZIKO coding scheme (Laevers, 2005). The ZIKO scheme describes a measurement for, among others, children's engagement. It is designed for child-task engagement in open classroom settings. We adapted the scheme to include specific cues for this experiment by including cues such as, attention toward the experiment leader instead of the robot or tablet and child is randomly clicking on the tablet in order to continue.

Each engagement level had specific cues for the rater to look for. For example, children scored high on task-engagement when they were not only looking at the task and robot, but also actively searching for the different animals on the tablet and were fully committed to the task. In contrast, when children turned away from the robot and task, did not perform anything related to the task and were fiddling, this resulted in a low engagement. Children who played the game but did not pay all their attention to it received an average task-engagement rating. In the case of robot-engagement we added social engagement cues, such as looking at the robot, having spontaneous conversations with the robot, but it also included the children's posture toward the robot (a closed posture indicating a low robot engagement and an open posture indicating a high robot engagement).

For all specific cues and information, see the coding scheme in the Supplementary Material and on GitHub^3^.

For the engagement coding, we pseudo-randomly selected half of the children, excluding children who took a break during the interaction (for example when they had to go to the bathroom), which happened in 11 cases. Twenty percent of the selected data was coded by two raters and their inter rater agreement was considered moderate to good using the intraclass correlation coefficient (ICC_*task*_ = 0.70, 95% CI[0.37, 0.76], ICC_*robot*_ = 0.80, 95% CI[0.62, 0.90]) (Koo and Li, 2016). For analyses, we only used the data of the first rater. We extracted two 2-min video fragments of the interaction: one at the beginning of the training rounds during the interaction and one at the end of the interaction.

The engagement rating of both fragments were combined to get a more reliable measure of the child's overall engagement during the lesson. This resulted in 210 engagement ratings in total.

#### 3.7.2. Analyses

To investigate the effect of the different feedback strategies on children's engagement, we conducted a repeated measures ANOVA with the feedback strategy as the independent variable (three levels) and engagement as a dependent variable.

In addition, to investigate the effect of the feedback strategies on learning gain, we carried out a two-way repeated measures ANOVA with the children's scores as a dependent variable and two strategies: (1) feedback strategy (three levels) and (2) test moment (the pre-test and the delayed post-test).

Using planned contrasts, we compared the effect of preferred and dispreferred feedback with no feedback on engagement and learning gain for H1 and preferred feedback and dispreferred feedback for H2. Moreover, to investigate the effect of the feedback strategies on short-term learning gain, a one-way repeated measures ANOVA with feedback strategy as the independent variable and the results of the in-game test as the dependent variable was performed.

## 4. Results

We have made the data set for this experiment publicly available^4^. In this section we report the children's engagement and their learning gain during the sessions. In addition, we report on the possible relation between learning gain and the children's engagement. Children received positive feedback during all 24 rounds in the preferred feedback condition and on average 14.30 times during the dispreferred feedback condition.

### 4.1. Engagement

Table 4 shows the overall results of both engagement types for the different lessons and different conditions. Overall, task-engagement (*M*= 5.57, *SD* = 1.63) was slightly higher than robot-engagement (*M*= 5.12, *SD* = 1.85). The two engagement types were moderately correlated [*r*_(105)_ = 0.50, *p* < 0.01], indicating that they both measure a different type of engagement.

**Table 4:** Average task- and robot-engagement rating over time (SD).

**Feedback strategy**	**All lessons**		**Lesson 1**		**Lesson 2**		**Lesson 3**	
	**Task**	**Robot**	**Task**	**Robot**	**Task**	**Robot**	**Task**	**Robot**
Preferred	6.17 (1.43)	6.14 (1.74)	6.77 (1.25)	6.85 (1.70)	6.15 (1.72)	6.30 (1.60)	5.54 (1.16)	5.25 (1.62)
Dispreferred	5.26 (1.48)	4.47 (1.80)	5.06 (0.98)	4.00 (1.25)	5.18 (1.59)	5.18 (2.05)	5.46 (1.69)	4.00 (1.66)
No feedback	5.27 (1.82)	4.74 (1.58)	6.00 (1.83)	5.21 (1.76)	5.41 (1.38)	4.41 (1.20)	4.10 (1.79)	4.45 (1.67)
Overall	5.57 (1.63)	5.12 (1.85)	6.07 (1.57)	5.45 (1.95)	5.53 (1.57)	5.26 (1.81)	5.10 (1.64)	4.56 (1.69)

#### 4.1.1. Task-Engagement

**Figure 3:**
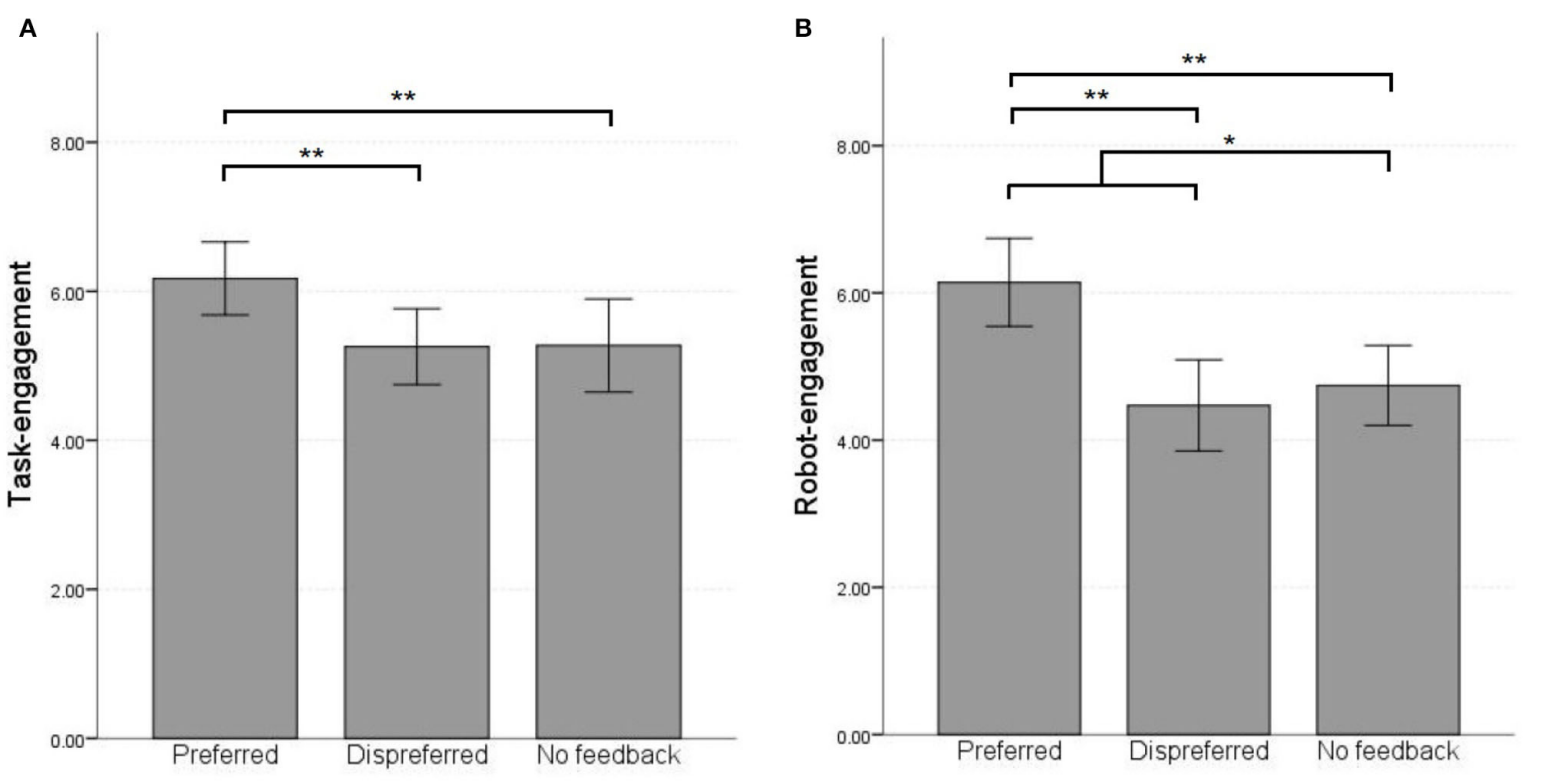
Average engagement ratings per condition. Error bars show 95% confidence interval. *p < 0.05, **p < 0.01. **(A)** Task-engagement. **(B)** Robot-engagement.

Contrary to our expectations, planned contrast analyses for comparing both preferred feedback and dispreferred feedback combined (*M* = 5.71, *SD* = 1.52) with no feedback (*M* = 5.27, *SD* = 1.82) showed no significant difference in task-engagement [$F_{\left(1,\;34\right)}=3.96,p=0.06,\eta_{p}^{2}=0.10$]. However, as Figure 3 shows, children are more engaged with preferred feedback (*M* = 6.17, *SD* = 1.43) than with dispreferred feedback [*M* = 5.26, *SD* = 1.48; $F_{\left(1,\;34\right)}=13.49,p=0.001,\eta_{p}^{2}=0.28$]. Further analysis using *post-hoc* comparisons with Bonferroni correction revealed that children were significantly more engaged in the preferred feedback condition than the no feedback condition [*t*_(34)_ = 3.26, *p* = 0.003, *M*_*diff*_ = 0.9]. There was no significant difference between dispreferred and no feedback [*t*_(34)_ = −0.06, *p* = 0.96, *M*_*diff*_ = −0.01].

**Figure 4:**
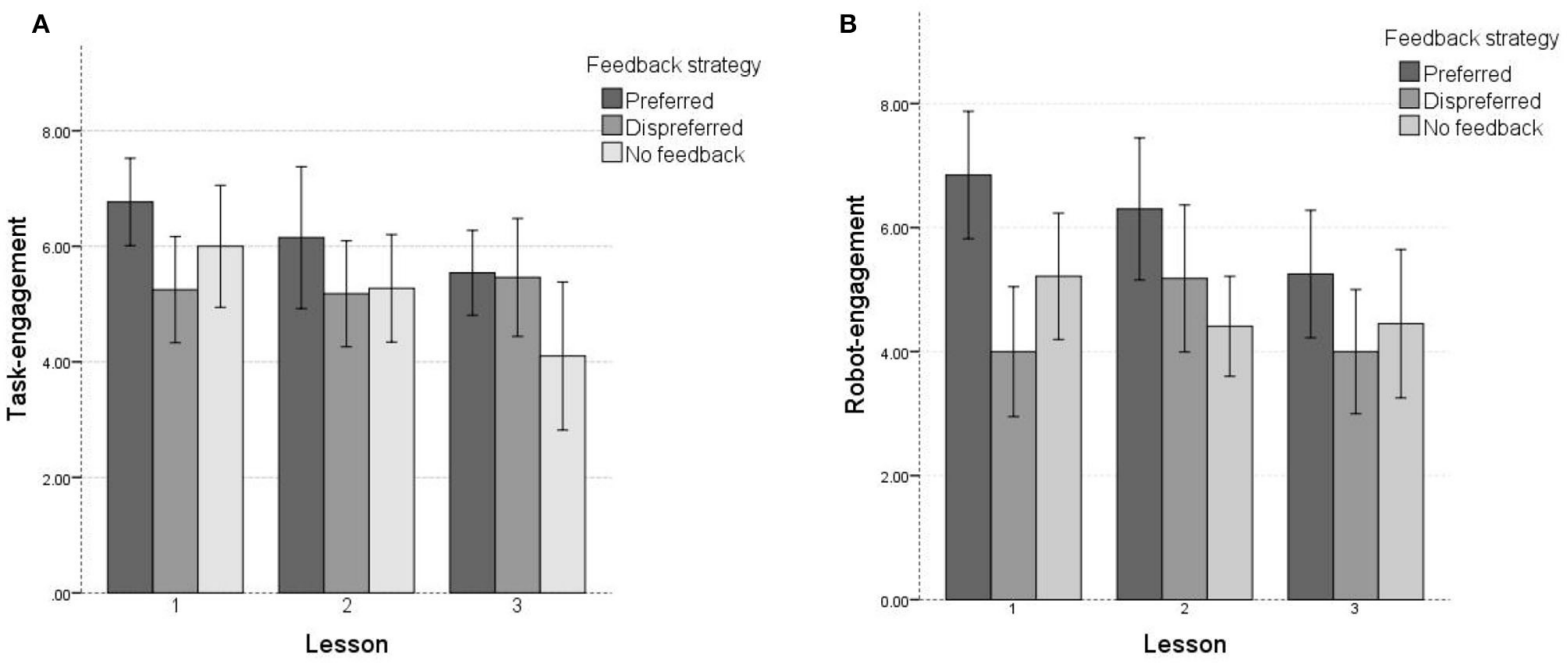
Average task- and robot-engagement ratings over time and per condition. Error bars show 95% confidence interval. Note that a child who, for example, received preferred feedback in lesson 1 received different feedback in lesson 2 and in lesson 3. **(A)** Task-engagement. **(B)** Robot-engagement.

Task-engagement dropped significantly over time (see Figure 4). A repeated measures ANOVA with a Huynh-Feldt correction was performed, because our data violated the assumption of sphericity. The analyses showed that task-engagement differed significantly between the lessons [$F_{\left(1.64,\;55.90\right)}=7.16,p=0.003,\eta_{p}^{2}=0.17$]. *Post-hoc* tests using the Bonferroni correction revealed that task-engagement dropped significantly between lesson 1 (*M* = 6.07, *SD* = 1.56) and 2 [*M* = 5.53, *SD* = 1.57; *t*_(34)_ = 2.82, *p* = 0.008, *M*_*diff*_ = 0.54], and lesson 3 [*M* = 5.10, *SD* = 1.64; *t*_(34)_ = 3.13, *p* = 0.004, *M*_*diff*_ = 0.97] but not between lesson 2 and 3 [*t*_(34)_ = 1.68, *p* = 0.102, *M*_*diff*_ = 0.43].

We further tested whether there was an interaction effect between the feedback strategy and the session in which it was used. To this end, we used a mixed ANOVA with order as between factor and feedback strategy as within factor, because this accounts for the order in which participants received the different feedback strategies (for example, it might have had an influence on their task-engagement when they received no feedback first and the preferred feedback during the third session). There was a significant interaction effect between order and feedback strategy [$F_{\left(10,\;58\right)}=4.43,p\;<\;0.001,\eta_{p}^{2}=0.433$] indicating that the effect of feedback on task-engagement varied as a function of when this feedback in the experiment it was administered taking into account that overall task-engagement decreased over time. As Table 5 illustrates, children's task-engagement dropped over time, but not for all orders of the feedback strategies. The task-engagement dropped in most situations after children received preferred feedback, task-engagement never increased after dispreferred feedback and it either dropped or remained the same for no feedback. An exploratory repeated measures ANOVA on each order indicated that task-engagement differed significantly when preferred feedback (*M* = 7, *SD* = 1.36) was provided first, then dispreferred feedback (*M* = 5.56, *SD* = 1.61) and lastly no feedback [$M=4.38,SD=1.85;F_{\left(2,\;14\right)}=18.11,p\;<\;.001,\eta_{p}^{2}=0.72$] and furthermore, when preferred feedback (*M* = 6.4, *SD* = 1.08) was provided first, then no feedback (*M* = 5.7, *SD* = 1.82) and lastly dispreferred feedback [$M=3.9,SD=1.82;F_{\left(2,\;8\right)}=8.11,p=0.012,\eta_{p}^{2}=0.67$]. All other orders did not differ significantly (all *p* > 0.1).

**Table 5:** The task-engagement order effects visualized, a decreasing arrow shows decreasing task-engagement and visa versa.

**Lesson 1**		**Lesson 2**		**Lesson 3**
**P**	↘	**D**	↘	N**
**P**	↘	**N**	↘	D*
D	→	P	↘	N
D	→	N	→	P
N	→	P	→	D
N	→	D	↗	P

P stands for preferred feedback, D for dispreferred feedback and N for no feedback. Task-engagement differed significantly for the first two orders with *indicating a p < 0.05 and **p < 0.01.

#### 4.1.2. Robot-Engagement

Similarly as for task-engagement, we compared the average children's robot-engagement score during both the feedback conditions (*M* = 5.31, *SD* = 1.95) with the no feedback condition (*M* = 4.74, *SD* = 1.58) using planned contrast analyses. Unlike for task-engagement, we found a significant difference in robot-engagement between feedback and no feedback [$F_{\left(1,\;34\right)}=4.39,p=0.044,\eta_{p}^{2}=0.11$], albeit with a relatively small effect size. Moreover, children scored higher for robot-engagement in the preferred feedback condition (*M* = 6.14, *SD* = 1.74) than in the dispreferred feedback condition [$M=4.47,SD=1.80;F_{\left(1,\;34\right)}=43.19,p<\;0.01,\eta_{p}^{2}=0.56$]. Furthermore, *post-hoc* comparisons with Bonferroni correction revealed that children were significantly more engagement in the preferred feedback condition than in the no feedback condition [*t*_(34)_ = 6.57, *p* < 0.01, *M*_*diff*_ = 1.40]. There was no significant difference between robot-engagement in the dispreferred feedback condition and the no feedback condition [*t*_(34)_ = 4.61, *p* = 1.0, *M*_*diff*_ = −0.27].

As Figure 4B showed, robot-engagement also dropped over time. A repeated measures ANOVA showed a significant difference between the lessons [$F_{\left(2,\;68\right)}=4.56,p=0.014,\eta_{p}^{2}=0.12$]. Again, note that the effect size is relatively small. Pairwise comparisons with a Bonferroni correction showed that robot-engagement dropped significantly between lesson 1 and 3 [*t*_(34)_ = 2.67, *p* = 0.04, *M*_*diff*_ = 0.99]. There was no significant difference between lesson 1 and lesson 2 [*t*_(34)_ = 0.87, *p* = 1, *M*_*diff*_ = 0.29] and lesson 2 and 3 [*t*_(34)_ = 2.27, *p* = 0.09, *M*_*diff*_ = 0.7].

Similarly as with task-engagement, we investigated whether there was an interaction effect between the feedback strategy and the lesson in which the feedback strategy was used. To test this, we used a mixed ANOVA with order as between factor and feedback strategy as within factor. For robot-engagement, there was no order effect [*F*_(10, 58)_ = 1.58, *p* = 0.14] which indicates that the children's robot-engagement was not influenced by different orders of feedback.

### 4.2. Learning Gain

**Figure 5:**
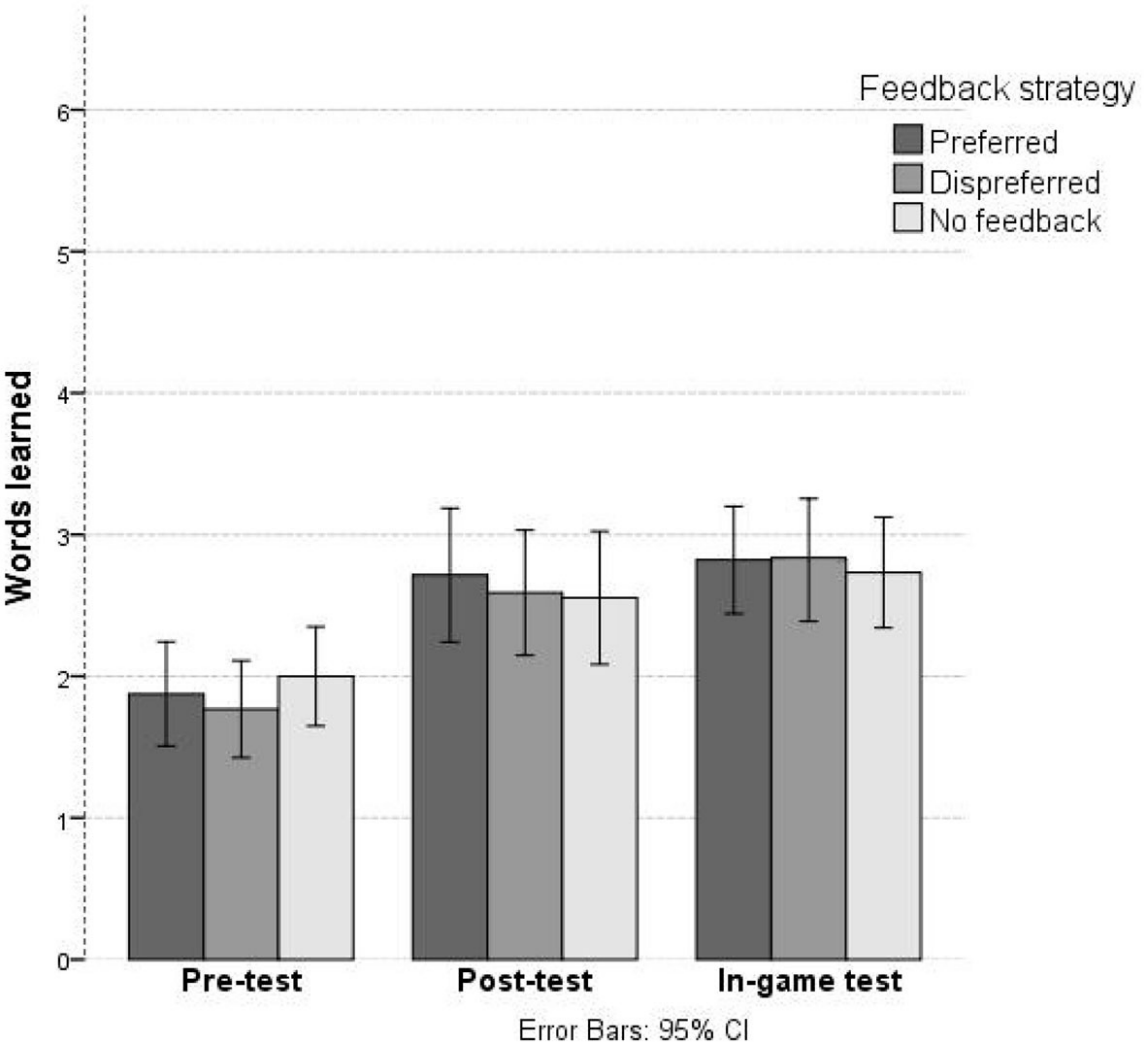
Learning gain per condition. Error bars show 95% confidence interval.

Children made on average 9.75 mistakes during the 24 rounds (Preferred: *M* = 9.95, *SD* = 5.56; Dispreferred: *M* = 9.30, *SD* = 5.22; No feedback: *M* = 9.75, *SD* = 5.41). Table 6 and Figure 5 show the descriptive statistics for the target word knowledge scores for all conditions. Children performed above chance level in the pre-test [chance level = 4.5, *t*_(55)_ = 4.27, *p* < 0.001, *M*_*diff*_ = 1.14] and post-test [chance level = 2.61, *t*_(55)_ = 9.58, *p* < 0.001, *M*_*diff*_ = 5.25]. As expected, children performed better on the post-test than on the pre-test [*t*_(55)_ = −3.88, *p* < 0.001, *d* = 0.52], so children clearly learned some vocabulary.

**Table 6:** Average score per condition (SD).

**Feedback strategy**	**Pre-test**	**Post-test**	**In-game**
Preferred	1.88 (1.38)	2.71 (1.77)	2.80 (1.42)
Dispreferred	1.77 (1.28)	2.59 (1.65)	2.82 (1.62)
No feedback	2.00 (1.31)	2.55 (1.76)	2.75 (1.43)
Total	5.64 (2.00)	7.86 (4.10)	8.38 (3.20)

The two-way repeated measures ANOVA with planned contrasts for both preferred feedback and dispreferred feedback (Pre-test: *M* = 1.82, *SD* = 1.33, Post-test: *M* = 2.65, *SD* = 1.70) showed no difference in learning gain compared to no feedback [Pre-test: *M* = 2.00, *SD* = 1.31, Post-test: *M* = 2.55, *SD* = 1.76);*F*_(1, 55)_ = 0.47, *p* = 0.83]. Furthermore, while children score numerically higher on word knowledge in the preferred feedback condition (Pre-test: *M* = 1.88, *SD* = 1.38, Post-test: *M* = 2.71, *SD* = 1.77) than in the dispreferred (Pre-test: *M* = 1.77, *SD* = 1.28, Post-test: *M* = 2.59, *SD* = 1.65), this difference was not significant [*F*_(1, 55)_ = 0.45, *p* = 0.51].

Table 6 also shows the results of the children's in-game tests. Children scored higher than chance in all conditions [chance level = 3, *t*_(55)_ = 12.57, *p* < 0.001, *M*_*diff*_ = 5.38]. Again, feedback strategy did not influence their learning gain, there were no significant differences [*F*_(2, 110)_ = 0.122, *p* = 0.89].

### 4.3. Relation Between Learning Gain and Engagement

To investigate whether there was a relation between both engagement types and learning gain, we performed a Pearson correlation analysis and in contrast with what we expected, we found no significant correlation between task-engagement and learning gain [Preferred: *r*_(35)_ = 0.05, *p* = 0.78, Dispreferred: *r*_(35)_ = 0.09, *p* = 0.62, No feedback: *r*_(35)_ = 0.12, *p* = 0.50]. Likewise, we did not find a significant correlation between robot-engagement and learning gain [Preferred: *r*_(35)_ = 0.15, *p* = 0.40, Dispreferred: *r*_(35)_ = 0.09, *p* = 0.62, No feedback: *r*_(35)_ = 0.02, *p* = 0.90].

## 5. Discussion

The aim of this study was to understand the effects that different types of robot feedback have on children's engagement both with the task, the robot and their learning gain. We derived different types of feedback from a survey with student teachers and implemented them in three different robots, each robot teaching children words from a second language in a single session. One robot provided (teacher) preferred feedback, one provided (teacher) dispreferred feedback, and one provided no feedback at all. All children attended three sessions, each with a different feedback strategy. We studied how this choice of feedback influenced children's task- and robot-engagement and their learning gains.

### 5.1. Engagement

The analyses of both engagement types suggest that children seem to be generally engaged with the task and the robot during the three sessions. This accords with human studies indicating that feedback can make tasks encouraging and engaging (Henderlong and Lepper, 2002).

Contrary to our expectations, when the robot provided feedback (either preferred or dispreferred), this did not lead to increased task-engagement compared to when the robot provided no feedback (H1a). Children who received no feedback were, on average, rated as equally engaged as children who did receive feedback. However, the type of feedback did seem to have an influence on task-engagement of the children: children became more engaged with a robot that provided preferred feedback than with one that used dispreferred feedback or indeed no feedback (H2a). Moreover, the robot's feedback did result into a higher robot-engagement compared to no feedback (H1a). Children who received feedback (either preferred or dispreferred), were rated more engaged with the robot than children who did not receive any feedback. However, it is worth pointing out that the numeric effects for robot- and task-engagement were rather comparable, even though the former but not the latter was found to be statistically significant. Similar to task-engagement, children were most engaged with a robot that provided preferred feedback (H2a) in comparison to dispreferred and no feedback. Interestingly, the difference between robot-engagement for preferred feedback and dispreferred feedback was larger than the difference for task-engagement.

Preferred and dispreferred feedback differed on multiple aspects (variation, extra attempt, repetition of answer, non-verbal behavior) and when combined, these factors seem to have an influence on engagement. While it is hard to identify exactly to what extent each of these factors contribute to children's task- and robot-engagement, we believe that some aspects might have had a larger effect on both engagement types than others.

For example, variation in feedback, as is realized in the preferred feedback condition, could have had relatively strong effect on children's task- and robot-engagement. A robot that provides more variation in the way feedback is offered could spark children's interest and keep them interested and motivated in continuing the task over a longer period of time and at the same time also make them more interested in the robot. In contrast, a robot who continually uses the same feedback phrase or no feedback at all might have a negative impact on children's interest in the robot and their robot-engagement and moreover reduce their motivation to continue with a task and, thus, be less successful in keeping them task-engaged.

It is furthermore possible that the extra attempt after an incorrect answer in the children's L1 may have task-engaged the children more in the preferred feedback condition than in the other two conditions. The fact that children heard the correct L1 word, could try again and received praise afterwards, may have had a positive effect on their task-engagement. This is in line with how teachers tend to provide feedback, praising demotivated children to try to engage them again (Hattie and Timperley, 2007). Some children also mentioned the extra attempt as the robot helping them getting the correct answer, this might increase their sense of relatedness to the robot which could have increased their robot-engagement.

Lastly, the non-verbal communication of the robot in the preferred condition may have increased children's robot-engagement as well. The robot displayed rotating colored eyes and nodded each time when children were correct. This is in agreement with the results of Morris and Zentall (2014), who found that children showed more intrinsic motivation when the robot used non-verbal behaviors such as thumbs up, and the findings of Serholt and Barendregt (2016), who found that children paid most attention to the robot when it provided feedback accompanied by an arm gesture. Future studies that take variation of feedback in combination with different types of non-verbal behavior into account will be needed to develop a full picture of this finding (De Wit et al., 2020). Besides gesturing, also gaze is a known non-verbal factor that can influence engagement (Mwangi et al., 2018). However, in the current experiment gaze was not factor of interest, since the robot's gaze behavior was identical in all three conditions.

As mentioned, it is not possible with the current experiment to determine which factor had the largest effect on task-engagement or robot-engagement. For this more research is needed. In the current experiment, we explored to what extent by student teachers preferred feedback strategy would differ from a dispreferred feedback strategy or no feedback strategy. We found that preferred feedback has a beneficial effect on both engagement types. However, to identify the effect of different factors that define the preferred feedback strategy has on engagement and which factor contribute to which engagement type, future experiments could be set up in which each factor is varied between conditions.

Also consistent with other studies is that both task- and robot-engagement seemed to drop over time (Kanda et al., 2007; Coninx et al., 2015; De Wit et al., 2018), and this drop appeared to be similar for all three conditions, although the differences between the conditions stayed over time. Adding more variation to the robot's feedback, as well as varying other parts of its behavior, might help reduce a drop in engagement. Ahmad et al. (2019) suggested that children seemed to stay engaged with a robot that is adaptive, which lends some support to the importance of individualized variation.

Interestingly, we found an interaction effect between task-engagement and the order of feedback strategies but not between robot-engagement and order. In particular, we observed that children's task-engagement dropped after receiving preferred feedback and that their task-engagement was similar or lower before receiving preferred feedback. Receiving no feedback or dispreferred feedback might have demotivated children, and, conversely, receiving various feedback information on their performance, might have increased their motivation again and therefore their task-engagement. Visa versa, after children received preferred feedback and continued in the dispreferred or no feedback condition, their task-engagement decreased again. However, some caution to this explanation must be applied, as the findings might have been influenced by individual differences as well.

### 5.2. Learning Gain

As expected, children learned from all three sessions with the robot. They did not learn many words per session though, which is in line with previous research with this young age group (Westlund and Breazeal, 2015; Vogt et al., 2019). Our results also show that these learning effects were retained in the longer run, because we conducted a post-test 2 weeks after the last session, suggesting that the target words remained in children's memory (Axelsson et al., 2016).

Contrary to our expectations, children did not learn more in the feedback conditions than when receiving no feedback (H1b), nor did it matter for the learning gain whether feedback was of the preferred or dispreferred variety (H2b). This was not only the case for the post-test, but also applied to the in-game test that was taken immediately after each training round.

What these results suggest is that children could learn from the teaching sessions without the need for feedback, and that the contribution of feedback to learning might have been smaller than we anticipated. This can be explained by the fact that children could rely on cross-situational learning (Smith and Yu, 2008), because children saw four depictions of possible meanings each time they heard a target word, with the distractors changing while the target stayed the same across situations. Hence, children could infer the meaning of a target based on the co-variation in meanings offered with the different occurrences of the target word, which seems to largely drive the learning, and feedback does not appear to contribute to this learning process.

It is conceivable that the learning task itself might have been too easy for the children to really benefit from the feedback. Moreover, since the children could press any animal they wanted to go forward in the game, they did not have to pay attention to the feedback of the robot. For future research, it would be interesting to conduct a study in such a way that feedback becomes more central to the interaction or more content-related, and where the learning task is more complex (e.g., learning about difficult sentence structures or unfamiliar grammar). This might shed further light on the influence of feedback on learning in child-robot interaction.

It is interesting to note that we did not observe learning differences between preferred and dispreferred feedback, which might be due to the feedback being completely offered in the children's L1. As a result, children did not receive a explicit translation between L1 and L2 as part of their (corrective) negative feedback. This might explain why children did not learn the L2 translation of a concept better during negative preferred feedback. It seems plausible that the addition of L2 to the negative (corrective) feedback would have resulted in higher learning gains (Hall, 2002; Scott and de la Fuente, 2008). However, we did not add this L2-L1 translation to our negative feedback for methodological reasons to keep the different conditions comparable. In particular, we made sure that there was an identical number of L2 exposures in every condition, since the number of L2 exposures could also affect learning (Ellis, 2002).

### 5.3. Relation Between Engagement and Learning

Various studies have found that increased engagement leads to better learning performance (Christenson et al., 2012). However, in our data we did not observe a relation between task- or robot-engagement and learning. Children who were more engaged with the task or with the robot did not learn more words than children who were less engaged. This might be due to the relatively small learning gain of children in the different conditions. They learned on average close to two out of six words during each session and this might not have been enough to observe a correlation with both engagement types. Moreover, it is conceivable that individual differences between children might have played a role as well. Effects of engagement on learning seemed to differ substantially from one child to the next, which is consistent with earlier research with this age group interacting with a robot (De Haas et al., 2017). Finally, we conjecture that in future research with more varied and more prominent feedback (along the lines sketched above), we might indeed observe that more engagement leads to better learning results.

### 5.4. Strengths and Limitations

This study has at least four strengths: First, we systematically compared different feedback strategies, derived from actual strategies suggested by young student teachers. Second, we tested a large group of young children to measure the effects of feedback. Third, the study was a carefully constructed experiment, of which all hypotheses and analyses have been preregistered (Simmons et al., 2011). Fourth, we measured two types of engagement to account for the children's engagement with the task and with their engagement with the robot as social partner.

Our study has also at least four limitations. First, we only measured comprehension and not active production of words. However, as speech recognition of the robot is not reliable yet, a more interactive task would have to rely fully on the experimenter in a Wizard of Oz setting (Kennedy et al., 2016). Since we aimed for an autonomously operating system, our task was designed to teach only passive understanding of L2 by using a tablet to record children's responses.

Second, our task was very repetitive. The only variation we introduced was the feedback that the robot would provide in the preferred feedback condition. Children did not have control over when to play with the robot and they were not able to change the task. It is a challenge to design a task that is adaptive to children's preferences, while still being educationally responsible and technical feasible. Providing such autonomy to children could increase their intrinsic motivation, which would increase their engagement and their learning performance (Ryan and Deci, 2000; van Minkelen et al., 2020).

Third, the robot could not react to the children's perceived engagement level during the experiment. While a human teacher would constantly monitor children's engagement and adapt the task accordingly to make it more personalized, the robot in our experiment simply continued to the next word and kept the interaction the same throughout all sessions, disregarding the child's engagement. Being able to automatically recognize a child's engagement would allow the robot to personalize feedback and other behaviors based on this engagement (Gordon et al., 2016; Ahmad et al., 2019).

Finally, we investigated the main effect of feedback on engagement and learning gain and showed that the preferred feedback had an influence on engagement with the task and with the robot. However, preferred and dispreferred feedback varied on multiple factors (variation, extra attempt, repetition of answer, non-verbal behavior), and consequently we cannot attribute the effect on engagement to only one of these factors, only the combination. Future research should look at individual aspects of feedback if technically feasible to measure the effectiveness for engagement.

## 6. Conclusion

The study presented in this paper explored whether robot feedback affects children's task- and robot-engagement and learning gain in second language learning. We compared three robot behaviors: one based its feedback on student teachers' preferred feedback strategies, one that did the opposite and one that did not use any feedback. The preferred strategy varied its feedback, gave children an additional attempt when they answered incorrectly, repeated the target word and gave non-verbal feedback. In contrast, the dispreferred feedback strategy did not vary its feedback, did not provide children with an additional attempt, did not repeat the target word and did not give non-verbal feedback. We found that children in the preferred feedback condition were more engaged than children in the dispreferred feedback and no feedback conditions, both with the task as with the robot. However, the feedback strategy did not influence children's learning gain; they did not retain more word knowledge with one of the different conditions. Moreover, we did not observe a relation between learning and engagement.

Our results are especially interesting for long-term interactions where engagement of children often drops. Providing feedback in an even more varied and motivating manner might help children to remain engaged in long-term scenarios. We expect that in the long-term such varied and motivating feedback can also improve children's learning gains, especially when the learning tasks become more difficult and children cannot just learn from inferring associations through cross-situational learning.

## Data Availability Statement

All datasets generated for this study are included in the article/Supplementary Material.

## Ethics Statement

The studies involving human participants were reviewed and approved by Research Ethics and Data Management Committee of Tilburg School of Humanities and Digital Sciences. Written informed consent to participate in this study was provided by the participants' legal guardian/next of kin.

## Author Contributions

MH, PV, and EK contributed to the conception and design of the study. MH developed, conducted, analyzed the study, and wrote the first draft of the paper. PV and EK supervised and critically reviewed the manuscript. All authors contributed to the article and approved the submitted version.

## Conflict of Interest

The authors declare that the research was conducted in the absence of any commercial or financial relationships that could be construed as a potential conflict of interest. The reviewer EB declared a past co-authorship with one of the authors MH to the handling Editor.

## Footnotes

^1^https://aspredicted.org/qg6dx.pdf

^2^https://github.com/l2tor/feedback-study/

^3^https://github.nl/l2tor/codingscheme. Please note that the coding scheme rates children on a scale of 1–5 including half points, which we have converted to a scale of 1–9 for convenience.

^4^https://doi.org/10.34894/ZEIKLY
