# Supplementary material for: The Effects of Feedback on Children's Engagement and Learning Outcomes in Robot-Assisted Second Language Learning
Source: Front Robot AI. 2020 Aug 4;7:101. doi: 10.3389/frobt.2020.00101 (PMC7806035; doi:10.3389/frobt.2020.00101)
Supplement: Supplementary file 1 [file Data_Sheet_1.pdf]

Protocol L2TOR coding engagement

This manual is based on an extensively tested measuring instrument called "ziko" (Laevers et al., 2005).
Before you can get started, you need to get to know 1 of the concepts behind the instrument: engagement.

It is important that you learn in advance to look specifically at children and know how to work with the instrument. You can only enter the scores correctly if you have mastered the manual. The preparation of the self-evaluation is of great importance. If you want more information and help with practicing ziko, go to ecego. You can find more information on the website of child and family (www.kindengezin.be) and of ecego (www.cego.be).

You can also find more information in these two publications:
Laevers, F. (2005). Well-being and Involvement in care settings. A process-oriented Self-evaluation Instrument (SIC's).
Laevers, F., Daems, M., De Bruyckere, G., Declercq, B., Moons, J., Silkens, K., ... & van Kessel, M. (2005). ZiKo. Zelfevaluatie-instrument voor welbevinden en betrokkenheid van kinderen in de opvang.

What is engagement?

A child who is engaged is, in a way, "completely absorbed" in his activity:
Playing with blocks, modelling clay or puzzling, listening to a story, talking to others, it is a very specific experience that you can recognize in both babies and adults.

Motivation

When you are engaged, you feel attracted by the activity, so you really are interested. You do not get engagement if others ask you or oblige you to do things. Your motivation arises from yourself, so although this may have been assigned to you, you are actively working on it yourself.

Intense mental activity

When you are engaged you find yourself completely open to experiences: the impressions you gain are intense. Body sensations and movement experiences, colours and sounds, smells and flavours have a hue and depth that would otherwise remain unnoticed. You use your imagination and cognitive ability fully.
In the absent of engagement, the sensations are not full lived through, that is, they are superficial.

Satisfaction

Engagement is a wonderful condition: you are ecstatic. What you experience is energy that passes through your body. Children spontaneously take initiatives that will keep them in that state. Playing is an excellent manner in which they find this satisfaction.

If engagement is lacking, you get bored, a feeling of emptiness and frustration.

Exploration urge

The source for engagement is the urge to explore, the urge to go around the world to gain sensory impressions, to get a grip on reality. Initially, that 'getting a hold' can be taken literally: touching and grasping whatever comes close. Gradually it is more about "understanding" reality.

At the limit of your capabilities

Engagement is possible if an activity is a challenge, neither too easy nor too difficult. When engaged, people move at the limit of their capabilities. They use their abilities to the fullest, they give the best of themselves - whether we are talking about babies or adults, or about children with poor mental development or about highly intelligent people.

What is engagement good for

Engagement is something very special. Anyone will get surprised by it, just by looking at children. You feel intuitively that you must not disturb the game. If there is engagement, we know that children are addressing their possibilities and that they are ‘developing ': they learn at a deeper level, and they become more competent.

Your job

You will determine the engagement of the child. Your method is simple and can be compared with 'scanning': you will observe the child for two minutes (1 video clip).
Give each child a score for engagement based on a five-point scale (1-5), where 1 is low engaged and 5 is highly engaged. You can also give half points, so the child can also be 3.5 engaged. When watching the video, remember that it is a snapshot, so it is possible that the same child scores low on engagement in one moment a higher in another moment.

Additionally, you look at engagement for the entire video clip. That means that if the child shows a higher engagement in the beginning of the clip compared to the last part; then you mediate the score of engagement over these two values. This mediating also depends on the period of time that the child shows this level of engagement, so for instance if the child shows a third of the video clip a high engagement (5) and shows a lower engagement (3) during two-thirds of the clip. Then, the final level for engagement will be between 3.5 and 4. Therefore, it is useful to make notes on how engaged the child is and why you think so.

We are going to measure two types of engagement: child task engagement and child robot engagement.

Measuring child-task engagement

Child-task engagement defines how the child is engaged with **the task.** A child can be engaged with a tablet, blocks in front of him/her, but also with the robot when the robot asks the child to do something (such as repeat and copy). If the child looks at the robot because the robot is talking, the child is still engaged in the task. Also, if the child looks at the robot when the robot shows a gesture, child task engagement remains. After all, repeating and gesturing are part of the task. Only in the case that the child focuses on something else during the task or looks at the robot for no reason do you score a lower level for the child task engagement. This also means that you do not measure how committed the child is with the robot, since that is the focus of the other engagement scale.

Child-task engagement is also accompanied by errors in the game; in general, a lower engagement leads to more errors by the child. But, as you probably recognize by conducting the experiments yourself, the system sometimes saw errors that were actually not wrong. In this case it is up to you not to include these errors in your child engagement score. **But always be aware that a low score on learning is not equal to a low engagement score, it is only possible that the two are related.**

Below you will find the scale for child-task engagement put in a table with examples.

Scale for child-task engagement		
Level	Engagement	Examples
**1**	Very low	The child shows virtually no activity:<br>• No concentration: staring, dreaming away;<br>• An absent, passive attitude;<br>• No targeted activity, aimless actions, not triggering anything;<br>• Meaningless ticking on the screen in order to continue;<br>• Only concerned with the experiment leader and not with the task;<br>• No signs of exploration and interest;<br>• Do not absorb anything, no mental activity
**2**	Low	The child shows some activity, but is regularly interrupted:<br>• Limited concentration: looking away, fidgeting, dreaming;<br>• Easily distracted;<br>• Tasks are performed to a limited extent
**3**	Mediocre	There is activity all the time, but not really concentrated<br>• The child is routine, fleeting;<br>• Has limited motivation, does not feel challenged, shows no real commitment;<br>• Does not gain in-depth experience;<br>• Is not absorbed by what it does;<br>• Only uses his capacities in moderation;<br>• The activity does not touch the imagination and the mind of the child.<br>• Most tasks are performed.
**4**	High	There are usually signs of engagement:<br>• The child is totally absorbed in his game;<br>• There is usually concentration, but sometimes the attention drops;<br>• The child feels challenged, there is a certain drive;<br>• Uses its abilities;<br>• Appeals to the imagination and the mind.
**5**	Very high	The child is continuously busy and becomes absorbed in his activity:<br>• Is continuously concentrated, absorbed by the activity, forgets about the time;

		• Is very motivated, feels strongly addressed;<br>• Cannot be distracted;<br>• Looks carefully at the task, pays attention to details;<br>• Is constantly appealing to all its capacities and possibilities;<br>• There is a strong mental activity: the imagination and the mind run at full speed;<br>• Gains profound new experiences;<br>• Enjoy being so passionately engaged.

Measuring child-robot engagement

Child-robot engagement only looks at how the child is engaged with **the robot**. This is not related to the task. The child can be engaged with the robot without the child performing the task. Child-robot engagement is determined by how often the child talks to the robot and looks at the robot. Only repeating a target word is not a sign of child-robot engagement, since the children in the tablet condition also talk after the tablet. If the child also looks at the robot when repeating the target word, it does count as child-robot engagement. Children who imitate the gestures of the robot also show a high engagement. A child who only looks at the tablet and ignores the robot (tries to ignore it) will score lower.

Scale for child-robot engagement

Level	Engagement	Examples
**1**	Very low	The child shows virtually no interaction with the robot:<br>• No concentration: staring, dreaming away;<br>• Ignores the robot completely;<br>• Has a closed (body) position towards the robot;<br>• An absent, passive attitude;<br>• No targeted activity, aimless actions, not triggering anything;<br>• No signs of interest in the robot.
**2**	Low	The child shows some interaction with the robot, but this is regularly interrupted:<br>• Limited concentration: looking away, fidgeting, dreaming;<br>• Limited looking at the robot;<br>• Easily distracted.
**3**	Mediocre	There is activity all the time with the robot, but not really concentrated.<br>• The child works routinely, being fleeting;<br>• Has limited motivation, does not feel challenged, shows no real commitment;<br>• Has an open (body) attitude towards the robot;<br>• Does not gain in-depth experience;<br>• Is not absorbed by the activity;<br>• Aimlessly touching the robot.

**4**	High	There are usually signs of robot-engagement:<br>• The child is totally absorbed in his game with the robot;<br>• There is usually joint attention;<br>• There is usually concentration, but sometimes the attention drops;<br>• The child feels challenged, there is a certain drive.
**5**	Very high	The child is continuously busy all the time and becomes absorbed in his activity with the robot:<br>• Is continuously focused on the robot;<br>• Feels strongly addressed; Cannot be distracted from the robot;<br>• Looks carefully at the robot, pays attention to details;<br>• Talks to the robot;<br>• Copies gestures;<br>• There is joint attention;<br>• Enjoy being so passionately engaged with the robot.
